# [Ca^2+^]_i_ oscillations in ASM: Relationship with persistent airflow obstruction in asthma

**DOI:** 10.1111/resp.12318

**Published:** 2014-05-22

**Authors:** David Sweeney, Fay Hollins, Edith Gomez, Ruth Saunders, RA John Challiss, Christopher E Brightling

**Affiliations:** 1 Institute for Lung Health, Department of Infection, Immunity and Inflammation, NIHR Biomedical Unit, Glenfield Hospital, University of LeicesterLeicester, UK; 2 Department of Cell Physiology and Pharmacology, University of LeicesterLeicester, UK

**Keywords:** airway hyperresponsiveness, airway smooth muscle, asthma, calcium, oscillations

## Abstract

The cause of airway smooth muscle (ASM) hypercontractility in asthma is not fully understood. The relationship of spontaneous intracellular calcium oscillation frequency in ASM to asthma severity was investigated. Oscillations were increased in subjects with impaired lung function abolished by extracellular calcium removal, attenuated by caffeine and unaffected by verapamil or nitrendipine. Whether modulation of increased spontaneous intracellular calcium oscillations in ASM from patients with impaired lung function represents a therapeutic target warrants further investigation.

Asthma affects over 300 million people worldwide. It is characterized by variable airflow obstruction (AFO) and airway hyperresponsiveness as a consequence of increased airway smooth muscle (ASM) contractility.^1^,^2^ There is emerging evidence that ASM from asthmatics is hypercontractile as demonstrated by an increased velocity of contraction in response to electrical field stimulation at the single cell level^3^ and in cell populations using gel contraction assays.^4^,^5^

Spontaneous ASM contraction is considered of fundamental importance in foetal lung development.^6^ Spontaneous intracellular calcium ([Ca^2+^]_i_) oscillations are reported in foetal ASM and are implicated in ASM contraction. Agonist-induced [Ca^2+^]_i_ oscillations are also reported in adult ASM and are implicated in augmenting ASM contraction and Ca^2+^-dependent transcriptional regulation.^7^,^8^ Spontaneous Ca^2+^ oscillations have not hitherto been reported in ASM from adults. We hypothesized that spontaneous [Ca^2+^]_i_ oscillations are maintained in ASM derived from subjects with asthma and are related to disease severity and disordered airway physiology.

Primary ASM was derived from bronchial biopsies obtained from well-characterized volunteers. All subjects gave written informed consent. The Leicestershire, Northamptonshire and Rutland Ethics committee approved the study. ASM cells were cultured and characterized as previously described^9^ and used at passages 2–5. To measure changes in [Ca^2+^]_i_, subconfluent ASM cells were loaded with 2 μmol/L Fura-2AM in the presence of 2.5 mmol/L probenecid and 0.04% w/v pluronic F-127, and visualized on an inverted epifluorescence microscope (Nikon Diaphot 200, Nikon Instruments, Kingston, UK). Fura-2 fluorescence (F) emission intensity at 510 nm was measured following excitation at wavelengths of 340 and 380 nm, and reported as a ratio, R = F_340_/F_380_, such that R is directly proportional to [Ca^2+^]_i_. [Ca^2+^]_i_ oscillatory behaviour was analysed using two methods. First, the [Ca^2+^]_i_ oscillation frequency was measured by a single-blinded observer counting the number of oscillations reaching a 340/380 ratio >10% greater than the baseline derived for each donor for 10 min. Second, the [Ca^2+^]_i_ oscillation dominant frequency (and its amplitude) were determined by fast Fourier transform (FFT) spectral analysis using software previously developed for MATLAB.^10^ The two methods were significantly correlated (r_s_ = 0.58; *P* = 0.0002). The FFT-derived Ca^2+^ oscillation dominant frequency data are reported here. The effect of removal of extracellular Ca^2+^, IP_3_ receptor inhibition (caffeine, 10 mmol/L) and voltage-gated L-type Ca^2+^ channel inhibition (1 μmol/L verapamil and 1 μmol/L nitrendipine) on [Ca^2+^]_i_ oscillations in highly oscillating cells were investigated.

Statistical analyses were performed using Prism version 6 (GraphPad, San Diego, CA, USA) and IBM SPSS version 20 (SPSS, Inc., Chicago, IL, USA). Data are presented as median (interquartile ranges) or maximum dominant frequency for the cells for an individual donor. Comparisons before and after pharmacological interventions used paired Student's *t*-tests, and across groups Kruskal–Wallis test and *post-hoc* pairwise comparisons using Dunn's test. Spearman rank correlation coefficients were used to assess the correlations. Previously, Ressmeyer *et al*.^7^ suggested that the oscillation frequency induced by histamine in ASM of lung slices relates to the degree of ASM contraction. Their work suggests that a dominant frequency of 60 mHz is equivalent to 30% contraction. We used this cut-off to identify the proportion of patients with cells that had dominant frequencies above this threshold. Proportions were analysed using chi-square test. A *P* value less than 0.05 was considered statistically significant.

We recruited 34 volunteers (14 healthy subjects (8 male, 6 female), 9 with mild-moderate asthma (4 male, 5 female) and 11 severe asthmatics (6 male, 5 female)). Subjects were age-matched: 60 (5) years healthy, 46 (5) years mild/moderate asthma and 48 (5) years severe asthma (*P* = 0.71). The spirometry measurements for all subjects (healthy, mild-moderate asthma Global Initiative for Asthma (GINA) I-III and severe asthma GINA IV and V respectively (mean (standard error of the mean)) were: forced expiratory volume in 1 s (FEV_1_)% predicted: 97 (3), 91 (8) and 77 (6) (*P* < 0.05); FEV_1_/forced vital capacity (FVC)%: 79 (2), 70 (3) and 64 (3) (*P* < 0.05). The dominant [Ca^2+^]_i_ oscillation frequency was determined for each cell (minimum 5 cells per donor). Spontaneous ASM [Ca^2+^]_i_ oscillations were observed in both health and disease. Example traces are shown (Fig. 1a). Spontaneous [Ca^2+^]_i_ oscillations were abolished by removal of extracellular Ca^2+^ (99.4% ± 0.4; data not shown). The amplitude of spontaneous [Ca^2+^]_i_ oscillations were attenuated by caffeine, being restored following its washout (*n* = 4 donors, 91 individual cells assessed; ANOVA *P* = 0.022; Fig. 1b) but was not significantly affected by verapamil or nitrendipine (data not shown). Spontaneous ASM [Ca^2+^]_i_ oscillation median dominant frequency was not different between asthma and health, or across disease severity (Fig. 1c). The maximum dominant frequency was increased in severe asthma compared with mild-moderate and healthy controls (*P* = 0.048 Kruskal–Wallis; Fig. 1d). Four of 11 severe asthmatic patients and none of the healthy subjects or patients with mild/moderate asthma had a maximum [Ca^2+^]_i_ oscillation dominant frequency greater than 60 mHz (*P* = 0.009; Fig. 1d). There were no differences in the amplitude of the dominant frequency across groups (data not shown). Interestingly, the median [Ca^2+^]_i_ oscillation dominant frequency was significantly correlated with FEV_1_% predicted (r_s_ = −0.54, *P* = 0.014) and with FEV_1_/FVC% (r_s_ = −0.68, *P* = 0.0009; Fig. 1e) in those with asthma. Furthermore, in those asthmatics with persistent AFO (FEV_1_% predicted <80% and FEV_1_/FVC <70%) compared with those without persistent AFO the median [Ca^2+^]_i_ dominant oscillation frequency was significantly increased (5.17 (4.5–7.4) vs 2.4 (2.0–3.7) mHz), *P* = 0.001; Fig. 1f). Similar observations were made between oscillation frequency determined by >10% greater than baseline and lung function (correlation FEV_1_/FVC% and oscillation frequency *r* = −0.47, *P* = 0.005; other data not shown).

**Figure 1:**
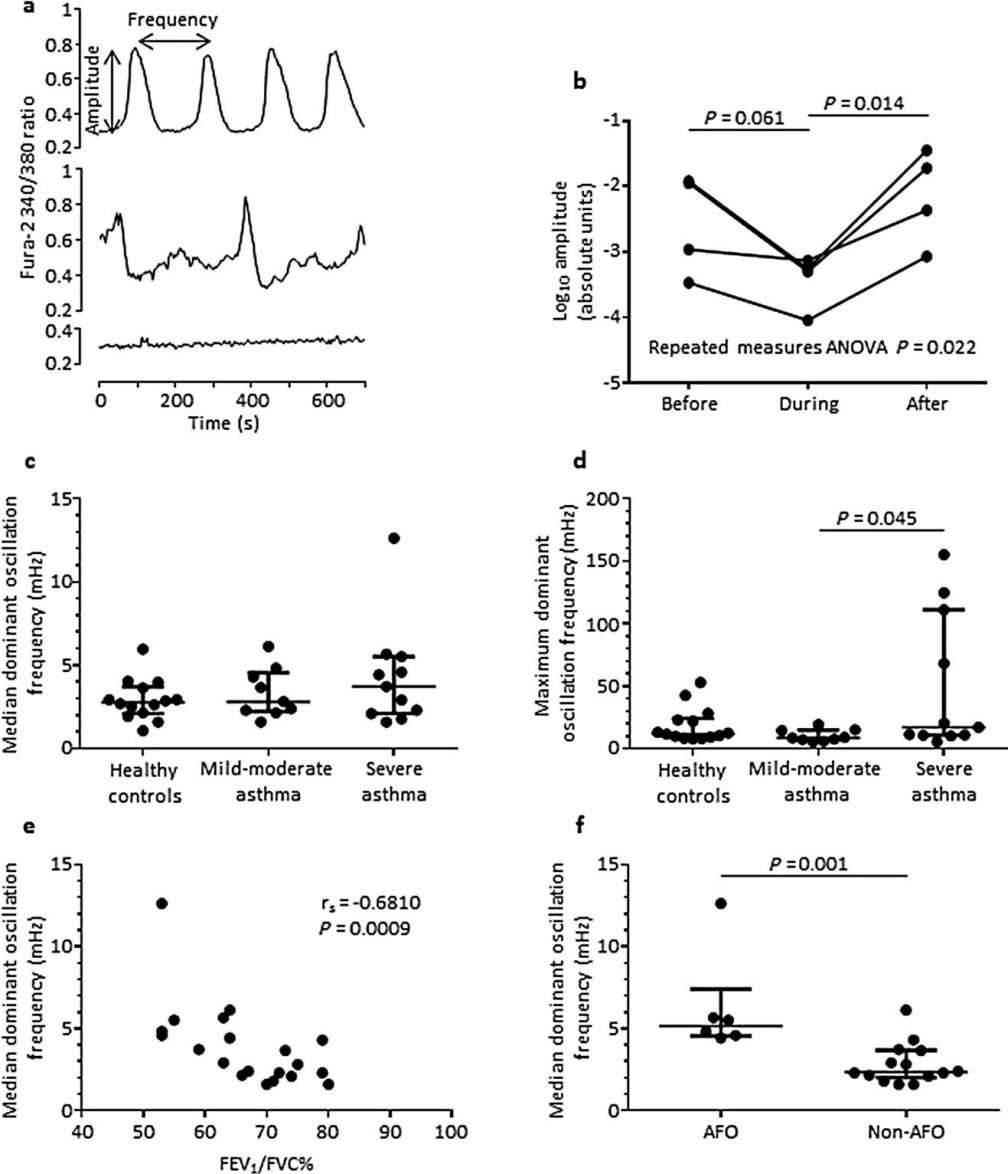
(a) Examples of intracellular calcium ([Ca^2+^]_i_) traces showing a low oscillating cell, a pronounced oscillating cell with high amplitude, regular frequency and little background, and an oscillating cell with higher background interference. (b) Median amplitude of [Ca^2+^]_i_ oscillations before, during and after caffeine (10 mmol/L) application for four donors derived from 91 cell traces. Repeat measures analysis of variance (ANOVA) and pairwise *t*-test *P*-values are as shown. (c) Median dominant oscillation frequencies of subjects with asthma were compared with healthy controls. Each point is an individual subject. Horizontal bars represent medians. (d) Maximum dominant oscillation frequencies of subjects with asthma were compared with healthy controls. Each point is an individual subject. Horizontal solid bars represent medians. Comparisons made using Kruskal–Wallis test (*P* = 0.048) and *post-hoc* pairwise comparisons using Dunn's test as shown. (e) Median dominant oscillation frequency was correlated negatively with forced expiratory volume in 1 s (FEV_1_)/forced vital capacity (FVC)% Spearman's rank correlation coefficient. (f) Dominant oscillation frequency for asthmatic subjects with and without persistent airflow obstruction (FEV_1_% predicted <80%, FEV_1_/FVC <70%). Horizontal bars represent medians. Comparison between groups by Mann–Whitney test.

We report here for the first time that spontaneous [Ca^2+^]_i_ oscillations are observed in primary ASM cells in asthma and health. The maximum dominant frequency of these spontaneous [Ca^2+^]_i_ oscillations was increased in severe asthma. The median dominant frequency was related to impaired lung function and was increased in asthmatics with persistent AFO. Agonist-induced [Ca^2+^]_i_ oscillations have been consistently observed, and previous reports have primarily implicated the intracellular Ca^2+^ stores.^7^,^8^ However, other reports have implicated both intracellular Ca^2+^ stores and influx pathways.^11^ Altered Ca^2+^ homeostasis has previously been reported as a consequence of altered mitochondrial biogenesis^12^ and SERCA2 downregulation,^13^ suggesting that intracellular Ca^2+^-handling in ASM in asthma might be altered via a number of interrelated mechanisms. The consequences of spontaneous [Ca^2+^]_i_ oscillations in asthma is not fully understood but have been implicated in increased ASM contraction and Ca^2+^-transcriptional coupling.^7^,^8^ Indeed, agonist-induced ASM hypercontractility is reported in primary ASM from asthmatics.^4^,^5^ Ressmeyer and colleagues demonstrated a relationship between the agonist-induced [Ca^2+^]_i_ oscillation frequency and percentage airway contraction in human airway slices.^7^ The frequency of the spontaneous [Ca^2+^]_i_ oscillations we describe here, predominantly in patients with severe asthma, might be sufficient to induce contraction and may also enhance the response to agonists. In addition to hypercontractility, primary ASM from asthmatics have increased capacity to release several important pro-inflammatory chemokines and matrix proteins compared with ASM derived from healthy volunteers.^5^,^14^ Although the potential role of spontaneous [Ca^2+^]_i_ oscillations in these mechanisms warrants further study, the associations observed here with asthma severity and disordered airway physiology suggests that these observations might be clinically important. The presence of spontaneous [Ca^2+^]_i_ oscillations in foetal ASM^6^ presents the intriguing possibility that this phenomenon might reflect either persistence or regression towards a foetal ASM phenotype in asthma. Whether this altered ASM is a consequence of the inflammatory effects of the asthmatic milieu or represents a mechanism independent of inflammation requires further study.

In conclusion, we have observed increased frequency of spontaneous [Ca^2+^]_i_ oscillations in ASM cells from subjects with impaired lung function. The mechanisms of this aberrant spontaneous [Ca^2+^]_i_ oscillatory behaviour in asthma need to be fully elucidated and might identify new therapeutic targets.

## Abbreviations

AFO: airflow obstruction
ASM: airway smooth muscle
[Ca^2+^]_i_: intracellular calcium
F: fluorescence
FEV_1_: forced expiratory volume in 1 s
FFT: fast Fourier transform
FVC: forced vital capacity
GINA: Global Initiative for Asthma

## References

[b1] Bousquet J, Mantzouranis E, Cruz AA, Aït-Khaled N, Baena-Cagnani CE, Bleecker ER, Brightling CE, Burney P, Bush A, Busse WW (2010). Uniform definition of asthma severity, control, and exacerbations: document presented for the World Health Organization Consultation on Severe Asthma. J. Allergy Clin. Immunol.

[b2] Global Strategy for Asthma Management and Prevention, Global Initiative for Asthma (GINA) (2011). http://www.ginasthma.org/.

[b3] Ma X, Wang Y, Stephens NL (1998). Serum deprivation induces a unique hypercontractile phenotype of cultured smooth muscle cells. Am. J. Physiol.

[b4] Matsumoto H, Moir LM, Oliver BG, Burgess JK, Roth M, Black JL, McParland BE (2007). Comparison of gel contraction mediated by airway smooth muscle cells from patients with and without asthma. Thorax.

[b5] Sutcliffe A, Hollins F, Gomez E, Saunders R, Doe C, Cooke M, Challiss RA, Brightling CE (2012). Increased nicotinamide adenine dinucleotide phosphate oxidase 4 expression mediates intrinsic airway smooth muscle hypercontractility in asthma. Am. J. Respir. Crit. Care Med.

[b6] Richards IS, Kulkarni A, Brooks SM (1991). Human fetal tracheal smooth muscle produces spontaneous electromechanical oscillations that are Ca^2+^ dependent and cholinerically potentiated. Dev. Pharmacol. Ther.

[b7] Ressmeyer AR, Bai Y, Delmotte P, Uy KF, Thistlethwaite P, Fraire A, Sato O, Ikebe M, Sanderson MJ (2010). Human airway contraction and formoterol-induced relaxation is determined by Ca^2+^ oscillations and Ca^2+^ sensitivity. Am. J. Respir. Cell Mol. Biol.

[b8] Savineau JP, Marthan R (2000). Cytosolic calcium oscillations in smooth muscle cells. News Physiol. Sci.

[b9] Kaur D, Saunders R, Berger P, Siddiqui S, Woodman L, Wardlaw A, Bradding P, Brightling CE (2006). Airway smooth muscle and mast cell-derived CC chemokine ligand 19 mediate airway smooth muscle migration in asthma. Am. J. Respir. Crit. Care Med.

[b10] Uhlén P (2004). Spectral analysis of calcium oscillations. Sci. STKE.

[b11] Hamada H, Damron DS, Hong SJ, Van Wagoner DR, Murray PA (1997). Phenylephrine-induced Ca^2+^ oscillations in canine pulmonary artery smooth muscle cells. Circ. Res.

[b12] Trian T, Benard G, Begueret H, Rossignol R, Girodet PO, Ghosh D, Ousova O, Vernejoux JM, Marthan R, Tunon-de-Lara JM (2007). Bronchial smooth muscle remodelling involves calcium-dependent enhanced mitochondrial biogenesis in asthma. J. Exp. Med.

[b13] Mahn K, Hirst SJ, Ying S, Holt MR, Lavender P, Ojo OO, Siew L, Simcock DE, McVicker CG, Kanabar V (2009). Diminished sarco/endoplasmic reticulum Ca^2+^ ATPase (SERCA) expression contributes to airway remodelling in bronchial asthma. Proc. Natl. Acad. Sci. U. S. A.

[b14] Alrashdan YA, Alkhouri H, Chen E, Lalor DJ, Poniris M, Henness S, Brightling CE, Burgess JK, Armour CL, Ammit AJ (2012). Asthmatic airway smooth muscle CXCL10 production: mitogen-activated protein kinase JNK involvement. Am. J. Physiol. Lung Cell. Mol. Physiol.

